# The Euthyphro Dilemma, Assisted Dying, and a Virtue Ethics Approach to Autonomy

**DOI:** 10.1111/bioe.70104

**Published:** 2026-03-22

**Authors:** Thomas Donaldson

**Affiliations:** ^1^ Centre for Social Ethics and Policy University of Manchester Manchester UK

## Abstract

The Euthyphro dilemma highlights that accounts of moral value which are dependent on the decisions of agents either result in arbitrary values arising from agent's decisions, or accept external reasons to morally justify the value, making the agent's decisions unnecessary for explaining the resulting value. This highlights an explanatory problem when the autonomous choices of patients form the basis of a moral justification for assisted dying. Providing any other moral reason in support of a request for assisted dying would undermine any moral justification for the request based solely upon the autonomy of the patient. When assisted dying is justified solely by patient's autonomous requests, patients requesting assisted dying are not treated as moral agents, because moral agents act for reasons. This does not mean that autonomy does not have a vital role in morality, as a safeguard against force, deceit or coercion being used to override the agency of patients. Autonomy is the necessary starting point of the development of virtue and the growth of human agency, but not its goal. In virtue ethics, patient's autonomous decisions, such as when requesting assistance to die, can be either virtuous or vicious, and so moral evaluation of the reasons for their choice is necessary to decide whether or not they can be shared by clinicians.

## 1 Introduction

The moral concept of autonomy has become increasingly important in liberal societies over the last few decades, but discussions of autonomy still have to overcome the challenges arising from the different definitions that the concept has been given. These include self‐governance, self‐expression, a Kantian adherence to principles of obligation towards other agents arising from universalising reason, and the right of a person to act upon their own judgement when deciding about matters of their own life, without interference from others [1]. The moral principle of respect for autonomy, describing a moral obligation not to interfere with a patient who is choosing to act in line with their own beliefs and values, has become a foundational moral consideration in clinical practice [2]. This principle highlights the importance of the patient's perspective, ensuring that patients are respected as agents and maintain control over their lives and bodies.

Despite the insistence of its authors [3], the widespread adoption of Beauchamp and Childress's four principles of biomedical ethics into medical practice and education in liberal societies has been interpreted to give autonomy pre‐eminence over other moral principles. This understanding of autonomy as the ‘first among equals’ [4] and as the overriding moral consideration in medical decision‐making, has resulted in autonomy‐based justifications of courses of action or treatment, to the exclusion of other moral considerations, and has been described as the ‘triumph of autonomy’ [5].

Arguments have been made for autonomy‐based approaches to assisted dying. For example, Petersen and Dige have argued, through their critique of arguments that requests for assisted dying are not autonomous, that assisted dying should be legalised because people can request assisted dying autonomously [6]. Braun has argued that a person's autonomous request for assisted suicide should be the only requirement for the provision of such assistance, because justifying such assistance by any other moral principle, such as beneficence, creates an expressivist objection where some lives are judged worth living and others not [7].

In a critique of the Netherlands' approach to assisted dying, Holzman has advocated that assisted dying should be available on an autonomy‐only basis, performed by a new non‐medical profession of end‐of‐life co‐ordinators, not doctors [8]. Holzman rejects the joint view of assisted dying decision‐making, where both the autonomous request of the patient and the clinician's judgement of unbearable suffering are required for assisted dying to occur [8]. She argues against the ‘unbearable suffering’ requirement, and the requirement that the suffering must be linked to a medical condition, as judged by a physician, because she considers it impossible for the physician's judgement of the subjective suffering of another to be objective. She also notes that the definition of medical conditions can evolve depending on social forces and influences, further limiting the potential of objectivity in physician judgements about the link between suffering and a medical condition [8]. For Holzman, autonomous requests for assisted dying should not be refused on the basis of physician judgements, as this would expose patients to the potential harm of wrongful refusals of assistance to die.

The autonomy of a decision to refuse assistance, for example, to refuse consent to a medical intervention, provides a moral reason against overriding it, even if it is an unwise, harmful, or otherwise bad decision. However, in cases where one agent is requesting assistance from another, the second agent is being asked to share the goal of the first agent and use their own agency to achieve that goal [9]. This means that both agents should, therefore, have a moral justification for seeking to achieve that goal. In cases where one agent is asking for assistance with achieving a harmful goal, such as being tortured, selling themselves into slavery, or selling their organs, the autonomy with which such a request is made would not normally be considered a sufficient moral justification for assisting with achieving that goal [10]. If this were the case, then purely autonomy‐based moral justifications of actions, where an autonomous decision is considered a sufficient condition for moral permissibility, would imply that autonomy or consent could justify any action [5]. However, a decision is not automatically considered morally right just because it was autonomously chosen [5]. Autonomy is necessary if humans are to act as agents, but human decisions can be good or bad [11]. Just like clinicians, patients also should be considered moral agents who make decisions for moral reasons [12], and the importance of having an explanation of moral values that is independent of the decisions of agents was explored by Plato.

## 2 The Euthyphro Dilemma

The Euthyphro dilemma arises from Plato's account of a dialogue between Socrates and Euthyphro about the nature of piety [13]. After Euthyphro attempts to define pious action as action that is loved by the gods, Socrates poses the question of whether an action is pious because the gods love it, or whether the gods love an action because it is pious. To avoid piety becoming dependent on the arbitrary whims of the gods, the interlocutors decide that the gods must love what is pious, which requires an alternative explanation of piety to be given [13]. Socrates then hints towards the possibility that piety could be defined in terms of justice.

This dilemma arising from this dialogue regards whether there is an asymmetrical explanatory relationship making piety dependent upon the approval of the gods, or whether the asymmetrical explanatory relationship occurs in the other direction, making the approval of the gods dependent upon piety [14]. The Euthyphro dilemma highlights an explanatory problem with making value concepts like piety and goodness dependent on divine decisions or attitudes. Even if all pious actions were also actions that were loved by the gods, without any category errors, this would not help explain what piety is, as the gods must love something because it is pious or else what is pious would be arbitrary. If divine attitudes and decisions about goodness and piety are based on reasons, then those reasons must appeal to a separate explanatory foundation, which is independent of the decisions and attitude of the gods. These reasons for an act being considered pious or good then become the explanatory basis for the act's piety or goodness, and the divine becomes unnecessary as an explanatory basis for concepts such as piety and goodness [15]. It has been argued that this dilemma was part of the way that Plato distinguished his ultimate ideal of the good from the divine [16]. Therefore, for Plato the will of the gods is understood to be dependent on the good rather than the good being dependent on the will of the gods [16].

However, the Euthyphro dilemma has potential applications beyond just theological relationships between the divine and concepts of piety and goodness. In this broadened scope, the Euthyphro dilemma has been applied to situations where a moral value is related to the decisions or attitudes of agents. If the moral value is explained by, or dependent upon, the decisions or attitudes of agents then the property has been described as Euthyphro subjective [17]. If the moral value is independent of, and not explained by, the decisions or attitudes of agents then the property has been described as Euthyphro objective [17].

This means that the Euthyphro dilemma can be applied to questions of morality in secular contexts. The secular version of the Euthyphro dilemma regards whether people's approval of an action explains its goodness (Euthyphro subjective), which would provide an account of moral motivation but would also seem to suggest that moral goodness is arbitrary. Alternatively, the goodness of an action could be independent of people's approval (Euthyphro objective), which means that goodness requires an alternative explanation and motivation [18]. A Euthyphro subjective understanding of morality finds the origin of goodness in human nature, basing moral valuing in human endeavours, within the intellectual and practical reach of human agents [17]. An Euthyphro objective morality (a realist morality) suggests a more aspirational morality, guiding humans to be better than their parochial interests and self‐serving projects [17]. It has been argued that the ideal morality would include the strengths of both Euthyphro subjectivity and Euthyphro objectivity [17].

This gives the Euthyphro dilemma relevance for constructivist accounts of morality. Constructivists argue that the correctness of the procedure for creating values is the ground for considering those values to be objective, not subjective [18]. However, the Euthyphro dilemma highlights a problem with explaining the normative basis for a moral constructivist procedure. If the moral construction procedure depends solely upon the attitudes and decisions of moral agents (a Euthyphro subjective construction procedure), then it is arbitrary which moral principles emerge from the construction process, and so anything could be considered good, even if it is out of keeping with our normal human moral commitments [15]. However, if the moral construction procedure is constrained or guided by moral principles (a Euthyphro objective construction procedure), then the constructivist will be covertly employing moral principles that exist independently of the construction process, which contradicts the fundamental goal of constructivism to develop a morality that is independent of pre‐existing moral principles [15]. An Euthyphro objective construction procedure could, therefore, be understood as a detectivist process (in line with moral realism), whereby moral values are created because they are right or wrong and the constructing agents are responding appropriately to this by discovering them through their construction procedure [15].

## 3 Preference Consequentialism and the Euthyphro Dilemma

At this stage in the argument, an important potential moral foundation for autonomy‐based approaches to assisted dying must be considered. This moral approach is preference consequentialism, which describes a group of moral theories that identify the good with the satisfaction of the preferences of the most possible people. An example of a preference consequentialist approach is Hare's arguments that harming a person is acting to prevent the satisfaction of their interests (or the realisation of their desires), where their interest is something they want, or a means to something they want [19]. This could lead to an argument that it would be good to assist someone with an autonomous decision to die, regardless of the reason that person may have for wanting to die. It could even imply that it would be wrong for a clinician to refuse to accept an autonomous request for assistance to die.

Savulescu has argued, from a consequentialist perspective, for the importance of respecting the choices of patients, even controversial ones [20]. However, when patients are making requests for medical interventions that could be harmful, such as the request to have their life ended, Savulescu has argued that clinicians have ‘an obligation to engage with patients requesting such care and examine their reasons for such care’ [20]. This duty is in keeping with the requirements of the Euthyphro dilemma, as examination of a patient's reasons for their decision, rather than just accepting their choice, is to treat them as a moral agent. If, upon examination of the patient's reasons for their request for assisted dying, the clinician judges that death is in their interests, then they can share the patient's reasons and offer assistance to end their life [21]. However, if the clinician judges that they cannot share the patient's reasons for their request, then this creates an argument against facilitating the patient's request and offering them assistance to end their life.

Without moral evaluation of a patients' reasons for their autonomous request, a clinician is not treating them as a fellow moral agent. Accepting a request for assisted dying without such moral evaluation is to treat the patient as a consumer whose desires must be fulfilled without moral consideration. Autonomy‐based justifications of assisted dying based on versions of preference utilitarianism that consider patient preference as the only moral consideration would not, therefore, be treating patients as moral agents. Part of treating patients as moral agents is the acknowledgement of the possibility that they can make bad decisions that should not be facilitated.

### 3.1 A Virtue Ethics Approach to Autonomy

Instead of considering autonomy as the sole basis of moral justifications of clinical actions, such that fulfilling patients' autonomous wishes becomes the goal at which clinical decision‐making must aim, autonomy is better understood as instrumentally valuable [22]. This is because an agent needs to have their autonomous decisions respected if their agency is to be effective. Human agency, understood in terms of ‘causal efficacy, of making a causal impact on the world and determining facts about it’ [23] cannot be made effective through force, deception, or coercion, only through choice. This provides the basis for respecting patients' autonomous decisions, even if the agent's decision is one that will itself hinder the effective realisation of their agency, because forcibly overriding an agent's autonomous decision will also not result in the effective realisation of their agency. Furthermore, the force required to override the decision of a competent agent may do them significant harm both physically and psychologically, which will result in additional restrictions on their agency and their flourishing [22]. The psychological damage involved in forcing a competent adult to receive an intervention that they do not want, is so significant it outweighs even the good of saving their life [9]. This means that autonomy should not be understood as a good in itself, but instead to derive its moral value from the intrinsic good of the effective realisation of human agency, which it defends.

For these reasons, autonomy is a vital foundation for virtue ethics because virtue must be chosen, and a virtuous character cannot be developed through force, coercion or deceit [24]. Respecting the autonomy of an agent is necessary to allow them to choose to start a process of virtuous character development through which they can grow into a fuller and more effective autonomy and agency. Virtue Ethics emphasises the importance of respect for autonomy as a pre‐condition for virtue and goodness, providing an important moral reason for respecting autonomous agent's decisions against coercion, deception, and forcefully being overridden. Therefore, it can be argued that this understanding of autonomy in virtue ethics has some of the key strengths of an Euthyphro subjective account of morality, highlighting the moral value of human choices and decisions.

However, in virtue ethics, something is not morally justified purely because it was autonomously chosen by an agent. Both virtue and vice can be autonomously chosen. Instead, moral justifications arise from consideration of the flourishing and promotion of the agency of an agent [25]. This *telos* (end or goal) acts as a guide for agents who have to consider whether to assist another agent with a particular action, based on a judgement regarding the moral value of their reasons for the action. This understanding of autonomy in virtue ethics avoids the explanatory problem of moral justifications based on the autonomous decisions of patients highlighted by the Euthyphro dilemma. Furthermore, a virtue ethics approach to autonomy also has the key strengths of an Euthyphro objective account of morality, because it provides an aspirational goal as a guide to agents. This means that a virtue ethics approach to autonomy can both explain why respecting the autonomy of patients is vital for virtuous development, whilst also providing a guiding *telos*, allowing the reasons and decisions of agents to be morally evaluated as either good or bad.

### 3.2 The Euthyphro Dilemma and Autonomy in Assisted Dying

Whilst the principle of respect for autonomy provides an important moral safeguard against violating a patient's agency or bodily integrity through the forceful, coercive, or deceptive implementation of treatment [26], the Euthyphro dilemma highlights an explanatory problem with the use of autonomy as the sole moral justification for action. If the patient's autonomous decision, for example, to request assisted dying, is sufficient moral justification to create an imperative on others to facilitate that decision, then no explanation for the moral justifiability of the action beyond the wishes of the patient is possible and morality becomes arbitrary, depending only upon what the patient says that it is. An autonomy‐based justification of assisted dying risks turning healthcare professionals into ‘hireling[s] of society’ [27], understanding clinicians as technicians or automatons rather than moral agents [28].

However, the Euthyphro dilemma highlights an even more significant problem with autonomy‐based justifications of assisted dying. They remove the possibility of patients providing a moral explanation of their reasons for requesting assistance to die. If patients offer moral reasons, independent of their autonomous request, then these reasons can be assessed by people who are not the patient. If the patient's reasons are judged to not support or justify the request for assisted dying, then this creates a moral reason not to accept the patient's autonomous request for that treatment. Instead, a patient's decision has to be understood as arbitrarily justified, impervious to external critique, but also excluded from moral defence and justification. Autonomy‐based justifications of assisted dying result in patients not being considered to be moral agents, who are able to give a moral account of their reasons for action, but instead considers patient to be consumers who can make use of assisted dying services for whatever reasons they choose, without moral consideration.^1^

The Euthyphro dilemma, therefore, highlights the problems inherent in the explanation of moral justification for action in terms of the decision and attitudes of agents. Requests for assisted dying require moral justification based in something more than the autonomous decision of an agent, if patients' reasons for these requests are to remain open to moral evaluation. This suggests that autonomy should not be understood as an intrinsic good in itself, because moral justifications arising solely from it must be considered arbitrary. Autonomy should rather be understood as an instrumental good, for example, within a virtue ethics framework, providing an important moral safeguard of an agent's autonomous decision‐making against deceit and coercion, which is necessary for agents to choose virtue and start to develop virtuous characters.

Applying a virtue ethics understanding of autonomy to assisted dying creates a counter‐argument to Holzman's view that the ‘unbearable suffering’ requirement should be removed because it gives the doctor *the* deciding power about assisted dying (my italics) [8]. In the Netherlands, the patient also has deciding power arising from the autonomy requirement, which means the patient's autonomous request is also necessary for assisted dying to occur. In the joint view of assisted dying decision‐making, both the patient and the doctor must be in agreement for the process to go ahead, and so both can stop the process occurring. Holzman's argument is analogous to saying the bride‐to‐be has *the* deciding power about whether a marriage goes ahead because it was the groom‐to‐be who proposed and the bride‐to‐be who said yes. However, marriages rely on both the bride‐to‐be and the groom‐to‐be being in agreement, regardless of who proposed the marriage and who agreed. This is the nature of any voluntary course of action requiring the involvement of more than one autonomous agent, such as assisted dying.

As a moral agent facing a decision about whether or not to assist someone to end their life, it is important that a clinician feels able to share a patient's reasons for wanting to die. An autonomy‐basis for assisted dying which removed the ‘unbearable suffering’ requirement would prevent clinicians from being able to evaluate a patient's reasons for requesting assisted dying and preventing clinicians from sharing these reasons with the patient. Even though the end‐of‐life represents the termination of a person's ability to develop a virtuous character and an effective agency, choosing to die does not necessarily exempt a person from virtue. There may be good reasons to choose to die, sacrificially saving another person being perhaps the most obvious example. However, there may also be bad reasons to choose to die, and this highlights the moral importance of considering the reasons for wanting to die when responding to a request for assisted dying.

## 4 Conclusion

Autonomy has a vital role in morality, as a safeguard against clinicians using deceit, force, or coercion to override the agency of patients. Autonomy is the starting point of the development of virtue and the growth of human agency, but the decisions of agents, such as choices to request assistance to die, must remain open to moral evaluation.

The Euthyphro dilemma highlights an explanatory problem with accounts of moral value or moral justifications for action that are dependent on the decisions of agents. Such accounts either result in arbitrary values arising from agents' decisions, which then become excluded from further explanation or justification, or require external reasons to morally justifying the value, making the decisions of agents unnecessary to explain and define the resulting value. This has implications for autonomy‐based justifications of assisted dying. When autonomy is the defining moral justification of assisted dying, the reasons why a patient has requested assistance to die are excluded from moral consideration. Such an approach does not treat patients as moral agents who could provide a moral account of their reasons and actions, but instead treats them as consumers who can make use of assisted dying services for whatever reasons they choose.

## Conflicts of Interest

The author declares no conflicts of interest.

## Endnotes

1 Autonomy‐based approaches to assisted dying could be considered analogous to making medications available on an over‐the‐counter basis, without prescription. Whilst this removes clinicians from involvement in decision‐making, the government or government agency responsible for making the drug available over‐the‐counter could be considered morally responsible for providing the assistance to die that would otherwise rest with the prescribing clinician. I thank the editor for this helpful comment.

## References

[1] G. M. Stirrat and R. Gill , “Autonomy in Medical Ethics After O'Neill,” Journal of Medical Ethics 31 (2005): 127–130, 10.1136/jme.2004.008292. 15738430 PMC1734107

[2] T. L. Beauchamp , “The ‘Four Principles' Approach to Health Care Ethics.” in Principles of Health Care Ethics, eds. R. E. Ashcroft , A. Dawson , H. Draper , and J. R. McMillan , 2nd ed. (Wiley, 2006), 3–10, 10.1002/9780470510544.ch1.

[3] T. L. Beauchamp and J. F. Childress , Principles of Biomedical Ethics, 6^th^ ed. (Oxford University Press, 2009), 99.

[4] R. Gillon , “Ethics Needs Principles—Four Can Encompass the Rest—And Respect for Autonomy Should be ‘First Among Equals’,” Journal of Medical Ethics 29 (2003): 307–312, 10.1136/jme.29.5.307. 14519842 PMC1733792

[5] O. O'Neill , Autonomy and Trust in Bioethics (Cambridge University Press, 2002), 47.

[6] T. S. Petersen and M. Dige , “Critique of Autonomy‐Based Arguments Against Legalising Assisted Dying,” Bioethics 37 (2023): 165–170, 10.1111/bioe.13125. 36417661 PMC10100019

[7] E. Braun , “An Autonomy‐Based Approach to Assisted Suicide: A Way to Avoid the Expressivist Objection Against Assisted Dying Laws,” Journal of Medical Ethics 49 (2023): 497–501, 10.1136/jme-2022-108375. 36190931 PMC10359509

[8] T. J. Holzman , “Creating a Safer and Better Functioning System: Lessons to be Learned From the Netherlands for an Ethical Defence of an Autonomy‐Only Approach to Assisted Dying,” Bioethics 38 (2024): 558–565, 10.1111/bioe.13296. 38712732

[9] T. Donaldson , “Suicide Booths and Assistance Without Moral Expression: A Response to Braun,” Journal of Medical Ethics 50 (2024): 718–720, 10.1136/jme-2023-109623. 37863648

[10] O. O'Neill , “Some Limits of Informed Consent,” Journal of Medical Ethics 29 (2003): 4–7, 10.1136/jme.29.1.4. 12569185 PMC1733683

[11] M. Friedman , “Autonomy, Social Disruption and Women.” in Relational Autonomy: Feminist Perspectives on Autonomy, Agency and the Social Self, eds. C. Mackensie and N. Stoljar (Oxford University Press, 2000), 35–51.

[12] A. Lelie and M. Verweij , “Futility Without a Dichotomy: Towards an Ideal Physician–Patient Relationship,” Bioethics 17 (2003): 21–31, 10.1111/1467-8519.00319. 12718331

[13] Plato , “Euthyphro,” in Plato: Complete Works, eds. B. Jowett (Amazon, 2020), 10a–15c.

[14] M. Macbeath , “The Euthyphro Dilemma,” Mind XCI, no. 364 (1982): 565–571, 10.1093/mind/XCI.364.565.

[15] C. Miller , “Euthyphro Dilemma,” in The International Encyclopedia of Ethics, eds. H. LaFollette (Blackwell, 2021), 1–7, 10.1002/9781444367072.WBIEE065.

[16] D. M. MacKinnon and H. Meynell , “The Euthyphro Dilemma,” Aristotelian Society Supplementary Volume 46, no. 1 (1972): 211–234, 10.1093/aristoteliansupp/46.1.211.

[17] K. Walden , “The Euthyphro Dilemma,” Philosophy and Phenomenological Research 90 (2015): 612–639, 10.1111/phpr.12055.

[18] J. Bojanowski , “Kant's Solution to the Euthyphro Dilemma,” Philosophia 44 (2016): 1209–1228, 10.1007/s11406-016-9747-2.

[19] R. M. Hare , “Wrongness and Harm.” Essays on the Moral Concepts (University of California Press, 1972), 92–109, 10.2307/jj.2430447.

[20] J. Savulescu , “Autonomy, the Good Life, and Controversial Choices,” in The Blackwell Guide to Medical Ethics, eds. R. Rhodes , L. P. Francis , and A. Silvers (Blackwell, 2008), 17–37, 10.1002/9780470690932.

[21] J. Savulescu , “Autonomy, Interests, Justice and Active Medical Euthanasia,” in New Directions in the Ethics of Assisted Suicide and Euthanasia, eds. M. Cholbi and J. Varelius (Springer International Publishing, 2015), 41–58, 10.1007/978-3-319-22050-5_4.

[22] T. Donaldson , “Human Flourishing, the Goals of Medicine and Integration of Palliative Care Considerations Into Intensive Care Decision‐Making,” Journal of Medical Ethics 50 (2024): 539–543, 10.1136/jme-2023-109299. 37945337

[23] T. Hurka , “Why Value Autonomy?,” Social Theory and Practice 13, no. 1987: 361–382, 10.1093/acprof:osobl/9780199743094.003.0008.

[24] Aristotle, *Nichomachean Ethics*, eds. D. Ross and L. Brown (Oxford University Press, 2009), 1112a1–1114b3.

[25] P. Foot , Natural Goodness (Oxford University Press, 2001), 39–68.

[26] E. C. Bullock , “Assisted Dying and the Proper Role of Patient Autonomy,” in New Directions in the Ethics of Assisted Suicide and Euthanasia, eds. J. Varelius and M. Cholbi (Springer, 2015), 1–16, 10.1007/978-3-031-25315-7_1.

[27] D. Callahan , “The Goals of Medicine. Setting New Priorities,” Hastings Center Report 26, no. 6 (1996): S1–S27, 10.1002/j.1552-146X.1996.tb04777.x. 8970793

[28] R. M. Veatch , “Models for Ethical Medicine in a Revolutionary Age,” Hastings Center Report 2 (1972): 5–7, 10.2307/3560825. 4679693

